# Advances in cervical cancer prevention: Efficacy, effectiveness, elimination?

**DOI:** 10.1371/journal.pmed.1003035

**Published:** 2020-01-28

**Authors:** Karin Sundström, K. Miriam Elfström

**Affiliations:** 1 Department of Laboratory Medicine, Division of Pathology, Karolinska Institutet, Stockholm, Sweden; 2 Regional Cancer Center Stockholm-Gotland, Stockholm, Sweden

## Abstract

With major advances in understanding the infectious etiology of cervical cancer, preventive medicine has obtained highly promising new tools. Human papillomavirus (HPV) vaccines, together with a growing arsenal of HPV-based screening tests, have the potential to radically change public health but require diligent, large-scale implementation to reach the final goal: the elimination of cervical cancer. We reflect here upon the state of cervical cancer prevention globally as there have been several recent developments that will inform this implementation process.

## Abstract 2

Karin Sundström and Miriam Elfström discuss prospects for and progress towards cervical cancer elimination.

## Evolution of HPV vaccination

Currently, there are 3 commercially available HPV vaccines: the bivalent (targeting high-risk types HPV16 and 18), quadrivalent (targeting HPV16, 18, and low-risk types 6 and 11) and nonavalent (targeting HPV6/11/16/18 and a further 5 high-risk types), which have all shown excellent efficacy against cervical cancer precursor lesions and, in the case of the latter 2, against external genital warts. In the future, we are likely to observe efficacy against large portions of the burden of HPV16-dominated anal, oropharyngeal, and penile cancers as well. Early in 2019, the United States Centers for Disease Communication and Control (CDC) increased the recommended upper age limit through 26 years for women and men to receive prophylactic HPV vaccination [1]. This followed an announcement made by the United Kingdom National Health Service (NHS) that free-of-charge HPV vaccination for boys aged 12–13 years would be provided from September 2019 [2]. These 2 landmark decisions can be seen as the natural continuation of a process in which the HPV vaccine has gone from being viewed as a “cervical cancer” vaccine, only for adolescent girls who have not made their sexual debut, to a vaccine that prevents infection with a virus transmitted throughout the life span that can also cause carcinomas in men [3].

## Prospects for cervical cancer elimination

A growing body of population-based studies have been key in shaping the globally accepted view of HPV vaccine outcomes, in which evidence of efficacy from randomized clinical trials (reviewed in [4]) is gradually complemented and refined by findings of sustained effectiveness in clinical practice (reviewed in [5]). In a similar development, early cost-effectiveness studies naturally focused initially on the impact on cervical outcomes of reaching high vaccination coverage among preadolescent girls [6]. However, it has since been proposed to extend vaccination to women up to age 30 or even 45–50 years [7], and subsequent modeling work has suggested that similar or greater benefits than those attained by vaccination of preadolescent girls may be achieved if vaccination is extended to older women and boys, if uptake is high in both sexes [8].

Such extended approaches would target the spread of the virus faster while at the same time providing direct cancer prevention in both women and men, and ambitions for disease prevention have grown accordingly. The growing evidence has suggested prospects for so-called disease elimination: when the rate of cervical cancer is reduced to a minimum and is no longer considered prevalent in the population—currently defined as a rate below 4 cases/100 000 woman-years [9]. In 2018, WHO issued a global call for cervical cancer elimination [10], in which disease containment/control is no longer the main goal but rather the decisive removal from circulation of one of the world’s major known carcinogens, as already projected in Australia, where a 20-year plan has been put in place and disease elimination is deemed possible [11].

## Opportunities and challenges posed by resource levels

The foundation for cervical cancer elimination is a 2-pronged approach in which vaccination in adolescents is complemented by more widespread cervical screening in women, defined in the motto “90:70:90”—i.e., 90% vaccinated, 70% screened, and 90% of those with cervical disease being offered effective treatment. Today, however, few regions worldwide reach near the ideal level of vaccinated girls. Despite carrying 80% of the global cervical cancer disease burden, typically because of an absence of effective screening at the population level, low- and middle-income countries (LMIC) in 2014 only accounted for 1% of the vaccinated girls worldwide [12]. For example, China only recently approved HPV vaccines for use, and coverage is low to nonexistent, especially in lower-resource regions. Indeed, the need for a more rapid rollout of vaccination in LMIC has been called for as the only way to reduce the risk for worsening global inequalities in prevention [12]. For this to be possible, vaccine delivery needs to be acceptable, available, and affordable. Yet, a systematic review recently found that there is a distinct lack of high-quality studies on HPV vaccine acceptability from these regions [13], and for 90% global coverage to be reached, such gaps in evidence need to be addressed.

Furthermore, although the logistical issues of cold chain storage and vaccine cost have long been anticipated, the most recent challenge that has arisen is a global limitation in the number of HPV vaccine doses available [14]. Timely delivery of vaccines, following international demand on the scale required for global viral elimination, could pose a challenge for manufacturers to meet. It follows that a key focus of future research should be estimating the effectiveness of 1 vaccine dose for children, because if fewer doses than the currently recommended 2 are enough, potential shortage issues would be alleviated. Naturally, a 1-dose schedule would itself lead to large public health gains: a 1-stop vaccination service, perhaps coordinated with cervical screening efforts, would suffice.

By the same logic, it should be equally valuable to show 2-dose vaccine schedule effectiveness in individuals greater than 13 years at age of vaccination, which—if firmly established—could allow a reduction from 3 doses to 2 and ensure that the available doses went even further.

Even in high-resource settings, elimination may prove a challenge, as exemplified by the situation in France, where HPV vaccine delivery has been challenging and uptake has remained low, at below 20% [15]. Experience from Sweden shows that, despite complete population registers and well-organized delivery, it is still challenging to achieve 90% coverage in population-based prevention programs [16]. Furthermore, the discrepancy in uptake of one preventive strategy may be exacerbated by lack of engagement in another: some girls are not vaccinated despite it being free of charge, and some women abstain from screening despite repeated, renewed invitations. Especially if combined, such choices lead to a particularly high risk for cervical cancer regardless of the overall resource level of the setting. If we do not reach these women, a substantial burden of cervical cancer will remain. Reaching out is now facilitated by HPV-based self-sampling kits, which have been shown to increase participation among previous screening nonattenders [17]. Self-sampling could be more broadly used to reach those for whom the standard model of clinician-based sampling is not acceptable or feasible.

## Cervical screening in the presence of HPV vaccination

Another programmatic challenge will be how to integrate vaccine services with cervical screening delivery, possibly in the HPV-FASTER concept, which suggests also vaccinating adult women against HPV (because this could eliminate HPV from the population faster while acknowledging that older, sexually active women’s benefit from the vaccine will be lower than that for adolescent girls) [7]. A further key issue will be how to adapt screening algorithms for increasingly vaccinated cohorts.

This is critical to achieve the greatest impact of prevention resources and reduce the burden on individuals of repeated vaccinations and screening rounds. Currently, the best evidence indicates that because the underlying probability of cervical lesions will diminish in vaccinated women, the positive predictive value of screening in such women will diminish as well [18,19]. It may thus be pertinent to consider HPV-based screening strategies specific to both age group and level of birth cohort HPV vaccine coverage, but this may not be trivial to resolve even for highly organized programs. Greater integration and the implementation of HPV-based screening, which has proven to be more effective in preventing cervical cancer than cytology [20], would position us well for achieving stronger preventive effects among groups of women not primarily targeted for vaccination.

## Future perspectives

Although efficacious strategies have been developed, several public health challenges remain in order to achieve effective global control of cancers driven by HPV. High-income countries should be able to achieve elimination more easily because of the presence of organized screening programs. Strengthening screening in LMIC will be paramount, e.g., through implementing primary HPV-based methods such as self-sampling and rapid HPV testing [21]. When it comes to vaccination uptake, however, we note that there are substantial differences between countries, not necessarily related to resource level. Indeed, several LMIC, including Rwanda and Bhutan, show excellent vaccine uptake of >90%, demonstrating that concerted efforts to match vaccination strategy with country-specific conditions can result in significant success independent of setting [22,23]. However, for elimination to occur, greater investments and constant vigilance will be required. Gavi, The Vaccine Alliance was successful in reaching its initial goal of 1 million vaccinated girls by 2015 in eligible countries, but the organization has recently stated that the target of 30 million vaccinated girls by 2030 is at risk [24].

The recent announcements from the US CDC, UK NHS, and WHO constitute important but incremental steps on the way to reduce cervical cancer. We must also keep in mind the time frame required to show sizeable reductions in disease, reductions that will take more or less time depending, firstly, on the success of the screening component of the elimination strategy—which, if successful, could detect and treat a large proportion of cervical cancer precursors before they come invasive, thereby leading to an early reduction in incidence of invasive cervical cancers—and secondly, on the time lag required between mass vaccination being performed and a preventive effect to be observed. Although much work remains, if complementary and equally ambitious measures in prevention program accessibility and adaptability are implemented and sustained, we believe that elimination of cervical cancer could move from prospect to reality.

## Abbreviations

CDC: Centers for Disease Communication and Control
HPV: human papillomavirus
LMIC: low- and middle-income countries
NHS: National Health Service
